# Revisiting the Mongolian Gerbil Model for Hepatitis E Virus by Reverse Genetics

**DOI:** 10.1128/spectrum.02193-21

**Published:** 2022-03-01

**Authors:** Ling-Dong Xu, Fei Zhang, Chu Chen, Lei Peng, Wen-Ting Luo, Ruiai Chen, Pinglong Xu, Yao-Wei Huang

**Affiliations:** a Department of Veterinary Medicine, Zhejiang University, Hangzhou, China; b MOE Laboratory of Biosystems Homeostasis & Protection, Zhejiang Provincial Key Laboratory for Cancer Molecular Cell Biology, Life Sciences Institute, Zhejiang University, Hangzhou, China; c Zhaoqing Branch Center of Guangdong Laboratory for Lingnan Modern Agricultural Science and Technology, Zhaoqing, Guangdong, China; University of Sussex

**Keywords:** hepatitis E virus (HEV), Mongolian gerbil, innate immunity, antivirals, interferon, reverse genetics

## Abstract

Hepatitis E virus (HEV) is a major cause of acute viral hepatitis in humans. A convenient small mammalian model for basic research and antiviral testing is still greatly needed. Although a small rodent, the Mongolian gerbil, was reported to be susceptible to swine genotype-4 HEV infection, whether the previous results were reliable and consistent needs to be validated by using biologically pure HEV stocks or infectious RNA. In this study, we revisited this gerbil infection model for human HEV of genotype 1, 3, or 4 (G1, G3, or G4) by HEV reverse genetics. Gerbils inoculated intrahepatically with capped G3 HEV RNA transcripts or intraperitoneally with infectious G3 cloned HEV produced robust infection, as evidenced by presence of HEV in livers, spleens, and feces for up to 7 weeks post inoculation, seroconversion, and pathological liver lesions. Furthermore, the value of the gerbil model in antiviral testing and type I IFN in host defense was assessed. We demonstrated the effectiveness of peg-IFNα-2a and ribavirin in inhibiting HEV replication in gerbils. By treatment with two molecule inhibitors of TBK1, we also revealed a role of RIG-I like receptor-interferon regulatory factor 3 in host anti-HEV innate immune sensing in this *in vivo* model. Finally, susceptibility of G4 HEV was demonstrated in intrahepatically inoculated gerbils with infectious HEV RNA transcripts, whereas no evidence for G1 HEV susceptibility was found. The availability of the convenient gerbil model will greatly facilitate HEV-specific antiviral development and assess the mechanism of host immune response during HEV infection.

**IMPORTANCE** HEV infects >20 million people annually, causing acute viral hepatitis as well as chronic hepatitis, neurological diseases, and pregnancy-associated high mortality, which require therapeutic intervention. The HEV antiviral research is largely limited by the lack of a convenient small animal model. Here we revisit the Mongolian gerbil model for three genotypes of human HEV by infectious HEV clones and recognized standards of experimental procedures. Fecal virus shedding, seroconversion, and pathological liver lesions could be detected in HEV-inoculated gerbils. We demonstrate the effectiveness and usefulness of this model in testing antiviral drugs, and in assessing the mechanism of host innate immune response upon HEV infection. This conventional rodent model will aid in future antiviral development and delineating mechanism of host immune response.

## INTRODUCTION

Hepatitis E virus (HEV) is the main pathogen of acute viral hepatitis. It causes large-scale hepatitis E (HE) outbreaks involving tens of thousands of individuals in developing countries with inadequate sanitation (1, 2). The mortality rate associated with HEV infection is less than 1%, but it can reach up to 25% in pregnant women (3). Recently, chronic HE cases have been reported in Europe and the United States, posing a particular threat to immunocompromised individuals including recipients of organ transplantations (4) and patients infected with human immunodeficiency virus (5). Human HEV is a quasi-enveloped virus with a single-stranded, positive-sense RNA genome of approximately 7.2-kb in size, which is classified into the species *Orthohepevirus A* of the family *Hepeviridae* (1, 2). HEV genome consists of a short 5′-untranlated region (UTR), three ORFs (ORF 1–3), and a short 3′-UTR followed by a poly(A) tract. The ORF1 encodes a large nonstructural protein with several functional domains including methyltransferase, de-ADP-ribosylation, helicase, and RNA-dependent RNA polymerase (RdRp) (1, 6). The ORF2 encodes a 660-amino acid (aa) capsid protein; more recently, its secreted form was identified with uncharacterized role (7). The ORF3 encodes a small 113-aa protein playing an important role in HEV morphogenesis and release *in vitro* (8, 9), and required for infectivity in monkeys and pigs (10, 11).

Thus far, four major genotypes (G1 to G4) of human HEV have been identified. G1 and G2 HEV are restricted to humans, and are commonly transmitted by the fecal-oral route via virus-contaminated water (3, 12). G3 and G4 HEV are zoonotic, and mainly transmitted by consumption of undercooked contaminated animal products (13). G3 has been identified from pigs, wild boars, rabbits, deer, and mongooses, whereas G4 has been predominantly found in domestic and wild swine (3, 14). The reverse genetic system, which allows direct genetic manipulation of RNA viruses, is one of the most important experimental platforms for HEV research (15, 16). A biologically pure form of a specific HEV strain generated from the full-length infectious cDNA clone is also required for analysis of a single phenotype since HEV is hardly isolated in cell culture from clinically positive samples (15–19).

However, studies on HEV pathogenesis, HEV-host interactions, and development of HEV-specific antiviral remain very challenging, partly due to the lack of a convenient small mammalian model. Laboratory mice or rats are not susceptible to human HEV infection. Animal models for studying HEV infection include pigs (16, 20), chickens (21), rabbits (22, 23), and nonhuman primates (15, 24). However, persistent HEV infection is difficult to achieve. Recently, a pig model and a rhesus macaque model for chronic hepatitis E with persistent infection have been developed, but these models require the frequent administration of immunosuppression drugs to animals (25, 26). A human liver chimeric mice model with persistent infection has also been developed, although it requires repopulation of mice with primary human hepatocytes (27).

The Mongolian gerbil (Meriones unguiculatus) is a small rodent that belongs to a species different from the more commonly used laboratory mice such as BALB/c (Mus musculus) or C57BL/6 inbred mice (28). Although several previous studies showed that infection of G4 swine HEV-positive liver samples or G1 human HEV-positive fecal samples led to detection of HEV antigens and lesions in gerbils (29–31), whether these results were reliable and consistent should be validated by using biologically pure HEV stocks and recognized standards of experimental procedures, which appeared lacking in these studies. In addition, whether G3 HEV is capable of infecting gerbils has not yet been determined.

In this study, we revisited and developed a gerbil model of productive G3 human HEV infection, either by intrahepatic injection with capped full-length viral RNA transcripts, or by intraperitoneal (i.p.) inoculation of infectious HEV generated by reverse genetics. We further illustrated the unique value of this model by demonstrating the usefulness of this model in testing antiviral drugs, and in assessing the mechanism of host innate immune response against HEV infection. Finally, we showed that, in addition to G3 HEV, gerbils are susceptible to G4 HEV, but not G1 HEV infection.

## RESULTS

### Intrahepatic inoculation of full-length capped RNA transcripts of a G3 human HEV (Kernow-C1 strain) infectious clone into gerbils leads to HEV infection.

**FIG 1:**
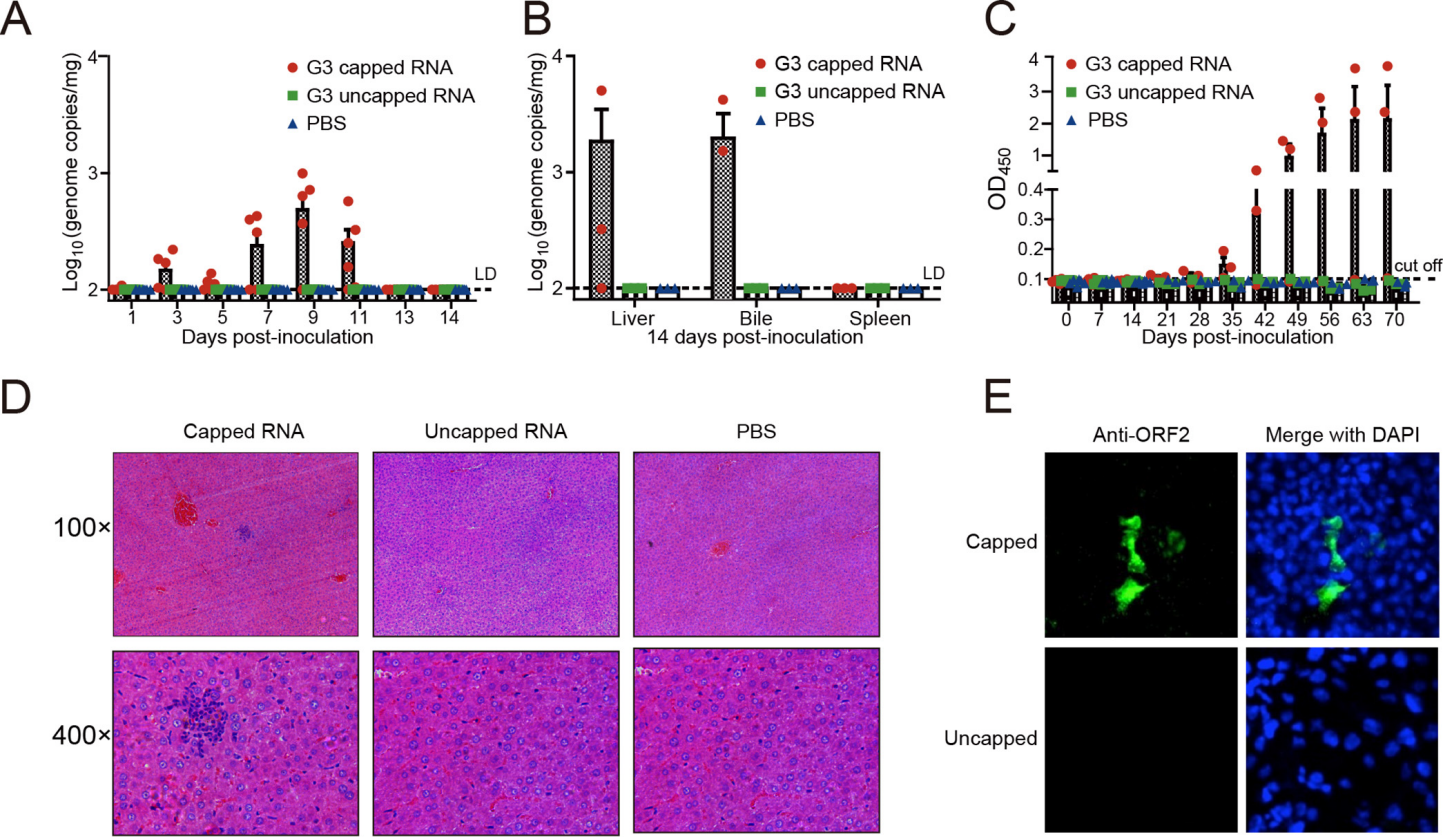
Successful establishment of a gerbil model for HEV infection via intrahepatic inoculation of capped HEV RNA transcripts from a genotype 3 (G3) human HEV (Kernow-C1 strain) infectious cDNA clone. Gerbils were inoculated intrahepatically either with PBS or uncapped or capped RNA transcripts from full-length HEV Kernow-C1 strain infectious cDNA clone p6. (A) Viral RNA in feces was determined by qPCR; *n* = 6 gerbils per group at each given time point. LD, limit of detection. (B) HEV RNA loads in liver, bile, and spleen were determined at 14 dpi by qPCR; *n* = 3 gerbils per group. Two out of four gerbils positive for fecal HEV shedding and one out of two HEV-negative gerbils were selected in the capped-RNA-inoculated group. (C) Seroconversion to anti-HEV IgG in infected gerbils was detected by ELISA with a corrected S/N ratio cutoff of 0.1 for positive samples. The error bars in all samples indicate standard deviation, *n* = 3 gerbils per group at any given time point. (D) Liver sections from gerbils intrahepatically injected with full-length capped or uncapped RNA transcripts or PBS, showing lymphocytic inflammatory infiltration in the capped RNA transcripts group. (E) Huh7-S10-3 cells were inoculated with homogenates of livers from gerbils that had been intrahepatically injected with capped or uncapped HEV RNAs (14 dpi). IFA was used to detect the expression of HEV ORF2.

Intrahepatic inoculation of HEV genomic RNA transcripts from an infectious cDNA clone has been routinely used to evaluate whether animals such as primates, pigs, or rats are susceptible to human or animal HEV infection (15, 16, 18). Herein, *in vitro*-transcribed capped and uncapped (as negative control) RNA transcripts from G3 human HEV Kernow-C1 P6 strain (17) were used to inoculate directly into the liver of gerbils (see “Animal experiment 1” in Materials and Methods for details). In four out of six animals receiving capped RNA transcripts, we detected fecal HEV shedding starting at 3 days postinfection (dpi), which peaked at 9 dpi (Fig. 1A). In contrast, we failed to detect fecal virus shedding in either of the negative control groups (uncapped RNA transcript or phosphate-buffered-saline [PBS]-inoculated groups) (Fig. 1A), further confirming the necessity of a 5′ cap structure in HEV genome for infectivity (15, 16). Quantification of HEV RNA in liver and bile from two out of four gerbils positive for fecal HEV shedding in the capped-RNA-inoculated group demonstrated a viral replication at 14 dpi (Fig. 1B), concomitant with seroconversion in the remaining two HEV-recovered gerbils by 21 dpi (Fig. 1C). Viremia was not detected in inoculated gerbils. Histological evaluation of liver sections from infected gerbils at 14 dpi showed the infiltration of lymphocytic inflammatory cells in the liver (Fig. 1D). In addition, HEV-specific antigen was detected by immunofluorescence assay (IFA) in Huh7-S10-3 liver cells inoculated with homogenates of liver collected from experimentally infected gerbils at 14 dpi (Fig. 1E). At the 10th week postinfection (wpi), HEV RNA was no longer detectable in the liver, bile or spleen of any of the groups. These data suggest that an establishment of the infected state following inoculation of capped RNA transcripts of a G3 human HEV clone is feasible in gerbils.

### Intraperitoneal inoculation of gerbils with infectious HEV leads to a robust and productive infection.

**FIG 2:**
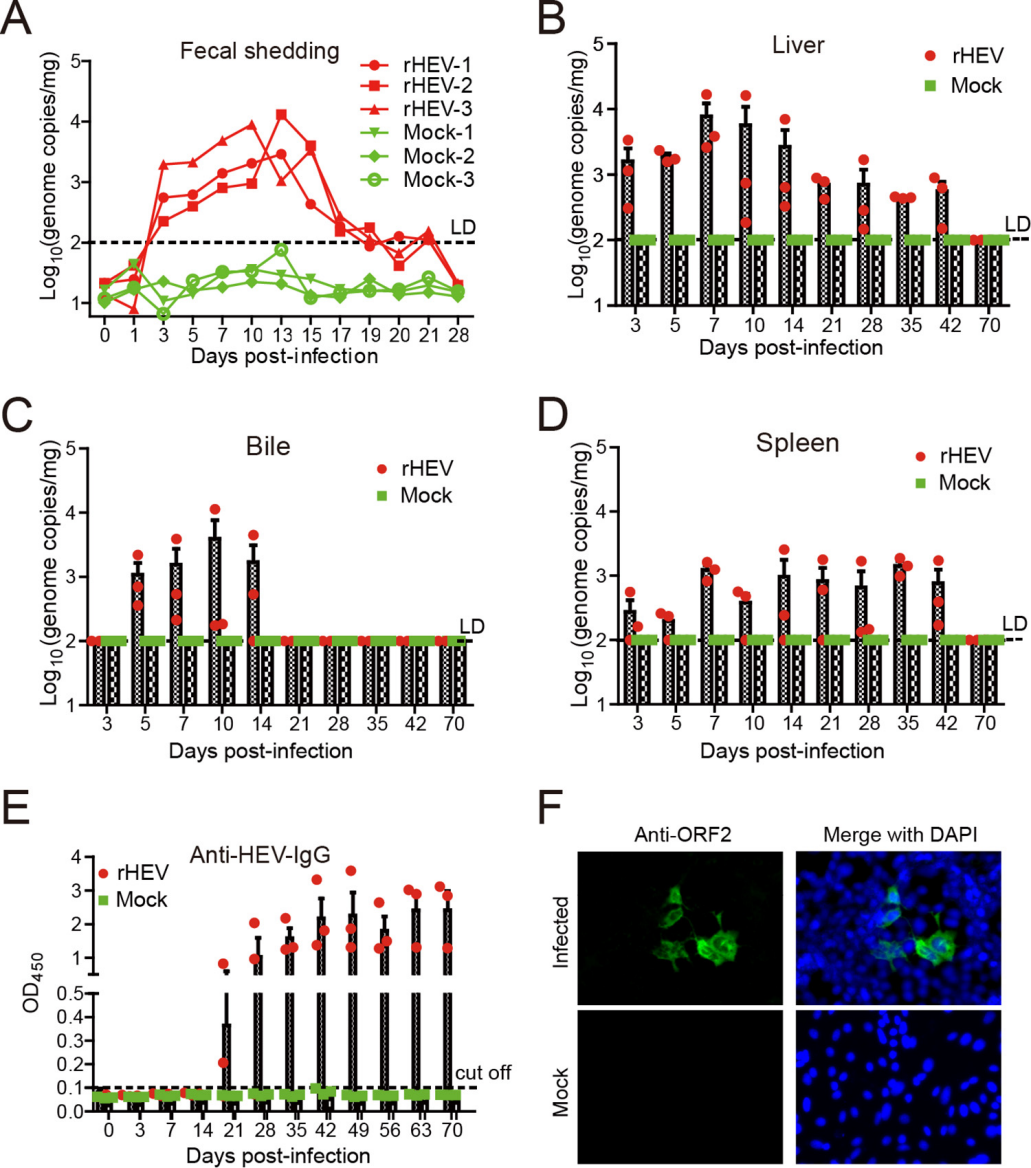
A gerbil model for robust HEV infection by intraperitoneal inoculation of infectious G3 HEV. Gerbils were inoculated intraperitoneally with infectious HEV stock rescued from the G3 HEV Kernow-C1 strain infectious cDNA clone p6 (rHEV). Virus titers (genome copies/mg tissue) in the (A) feces, (B) liver, (C) bile, and (D) spleen were measured at the indicated time points by qPCR; *n* = 3 gerbils per group at each time point. Fecal virus shedding in three gerbils was continuously monitored. (E) Anti-HEV IgG was detected by a commercial ELISA, using a corrected S/N ratio cutoff of 0.1 for positive samples. The error bars in all samples indicate standard deviation, *n* = 3 gerbils per group at each time point. (F) S10-3 cells were inoculated with homogenates of liver collected from infected gerbils at 14 dpi. IFA was used to detect the expression of HEV ORF2 antigen. In order to further confirm the presence of infectious virus, naive S10-3 cells were inoculated with homogenates of livers collected at 14 dpi from infected gerbils. Expression of HEV ORF2 was detected by IFA.

An intraperitoneal (i.p.) route of inoculation in rodents has been used to establish infection models for enteric viruses such as norovirus and coronavirus since it bypasses the interference of the intestinal acidic environment and mucosal immune barriers (32–34). To further demonstrate gerbils as a useful small animal model, infectious HEV stock was first produced by transfection of Huh7-S10-3 cells with HEV p6 capped RNA transcripts. Gerbils were then intraperitoneally inoculated with HEV stock (“Animal experiment 2”). HEV RNA was detected in the feces starting from 3 dpi, which peaked at 13 dpi with approximately 5.65 × 10^3^ copies/mg (Fig. 2A). Fecal viral RNA titers then decreased gradually and became undetectable at 28 dpi (Fig. 2A). HEV RNA was detected in the liver, bile, and spleen starting at 3 dpi and peaked at 10–14 dpi (Fig. 2B to D). Surprisingly, viral RNA titers in the spleen tissues remained constant up to 42 dpi (Fig. 2D). Seroconversion to IgG anti-HEV started at 21 dpi (Fig. 2E), concomitant with a rapid decrease of HEV RNA in the bile and feces, although the viral RNA titers decreased more slowly in the liver and spleen tissues. Moreover, IgG anti-HEV antibody in selected serum samples collected at 35 dpi was capable of neutralizing genotype 3 HEV *in vitro* (Table 1). The inability of neutralizing antibodies to quickly clear the virus from the liver and spleen at this time point was likely due to the presence of lipids in quasi-enveloped HEV virions to protect the particles from neutralizing antibodies in these sites (35), or was simply due to low neutralizing antibody titers at 35 dpi. Accordingly, the neutralizing antibody titers increased and consequently cleared the viruses at 70 dpi (Table 1 and Fig. 2B to D). HEV ORF2 antigens were detected by IFA in Huh7-S10-3 liver cells inoculated with homogenates of liver collected at 14 dpi from infected gerbils, thus confirming productive HEV infection (Fig. 2F).

**TABLE 1:** Neutralizing capability of selected gerbil serum samples on the infectivity of HEV Kernow-C1 p6 strain in S10-3 cells

Serum ID and controls	ELISA O.D. value^*a*^		% Decrease in HEV infectivity^*b*^		Neutralizing antibody titer	
	35^*c*^	70	35	70	35	70
PBS	N/A	N/A	0	0	N/A	N/A
Human-JS-1	N/A	N/A	87.42	78.11	N/A	N/A
Gerbil-1	1.256	1.283	54.29	55.57	1:8	1:16
Gerbil-2	1.312	2.841	51.43	56.23	1:16	1:64
Gerbil-3	2.412	3.114	58.1	61.77	1:32	1:64

a O.D., Optical density.

b All gerbil sera were tested in dilutions from 1:2 to 1:128 in a serum virus neutralization assay as described previously (57). The positive control sample Human-JS-1 is a convalescent phase serum with at least 10^4^ ELISA titer of anti-HEV IgG from a HEV-infected patient in Jiashan, Zhejiang province, China (58).

c Days post-infection (dpi).

**FIG 3:**
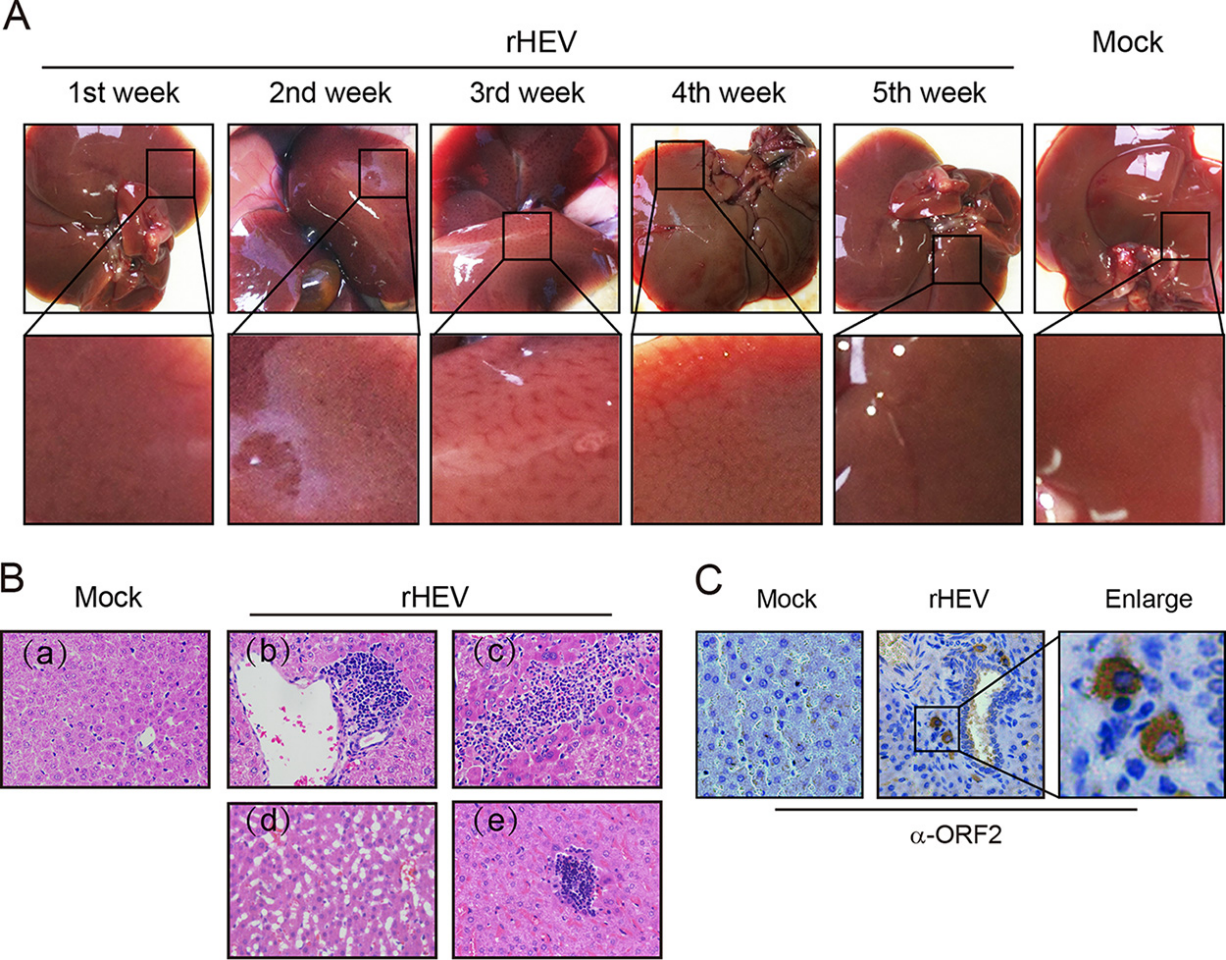
Gross and histopathologic lesions and immunohistochemistry (IHC) in the liver samples of G3 HEV-infected gerbils. (A) Representative gross pathological liver lesions from infected gerbils at 1–5 wpi. (B) Representative histopathologic lesions of the liver. Panel (a) is from a mock-infected gerbil; HEV-infected gerbils showed various lesions including (b) focally intense lymphocytic venous phlebitis and periphlebitis; (c) locally extensive patocellular necrosis with lymphocytic inflammatory cell infiltration; (d) necrosis and hydropic change of the hepatocytes; and (e) marked portal inflammatory reaction and congestion. (C) Paraffin-embedded gerbil liver sections were stained by IHC with anti-HEV ORF2 (brown signal).

Gross lesions were observed primarily in the liver, with increased vascular congestion in HEV-infected gerbils (Fig. 3A). The histological examination revealed lymphocytic periphlebitis and phlebitis foci in liver sections from 2–4 wpi (Fig. 3B, a and b). Other histological lesions include marked portal inflammatory reaction necrosis (Fig. 3B to c), hydropic change of the hepatocytes (Fig. 3B to d), and congestion (Fig. 3B to e) were also observed in the infected gerbils, consistent with an earlier report describing histological lesions in a chicken model for HEV (21). Furthermore, liver sections were stained positively by immunohistochemistry using anti-ORF2 antibody (Fig. 3C). The results demonstrate that gerbils are efficiently infected by genotype 3 human HEV and develop liver lesions after intraperitoneal inoculation.

### Oral inoculation of gerbils with infectious HEV does not result in efficient infection in each animal.

**FIG 4:**
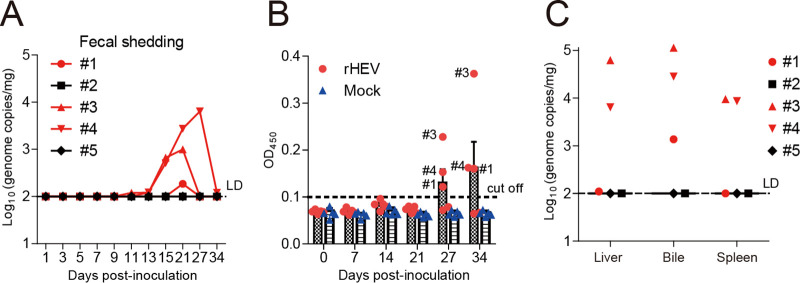
Oral injection of gerbils with G3 HEV had a less infection efficiency. Gerbils were inoculated orally with infectious HEV stock rescued from the G3 HEV Kernow-C1 strain infectious cDNA clone p6 (rHEV). (A) Viral RNA in feces was determined by qPCR, showing 5 gerbils (#1 to #5) in the HEV-inoculated group at each given time point. (B) Seroconversion to anti-HEV IgG in three out of five infected gerbils was detected by ELISA. The numbers of seropositive gerbils were labeled. The error bars in all samples indicate standard deviation. (C) HEV RNA loads in liver, bile, and spleen were determined at 34 dpi by qPCR, showing 5 gerbils (#1 to #5) in the HEV-inoculated group.

HEV is naturally transmitted via the fecal-oral route. In order to test whether gerbils could be infected orally, the animals were inoculated per orally (p.o.) at the same infectious dose as the i.p. route with pure HEV stock generated by the HEV p6 infectious clones (“Animal experiment 3”). In three out of five inoculated gerbils, we detected HEV RNA in fecal samples starting at 11, 13, or 21 dpi and lasting for 1 to 2 weeks (Fig. 4A), which appeared late compared to what was detected by the i.p. route. These three infected gerbils also had a delayed seroconversion at 27 dpi (Fig. 4B). At 34 dpi, HEV viral RNA was detected in the bile samples in each of three infected gerbils, whereas the liver and spleen samples had detectable HEV RNA in two out of three infected animals (Fig. 4C). Histological evaluation of liver sections from infected gerbils at 34 dpi displayed mild infiltration of lymphocytic inflammatory cells (data not shown). The remaining two inoculated gerbils and the control gerbils (fed with cell culture medium) had neither detectable viral RNA in the collected samples nor seroconversion (Fig. 4B and C). Therefore, the p.o. route is less efficient than the i.p. route for HEV infection in the gerbil model, and the latter was used as the standard procedure in the drug-trial study.

### The gerbil animal model offers a powerful tool for testing HEV antivirals (ribavirin and peg-IFNα-2a).

**FIG 5:**
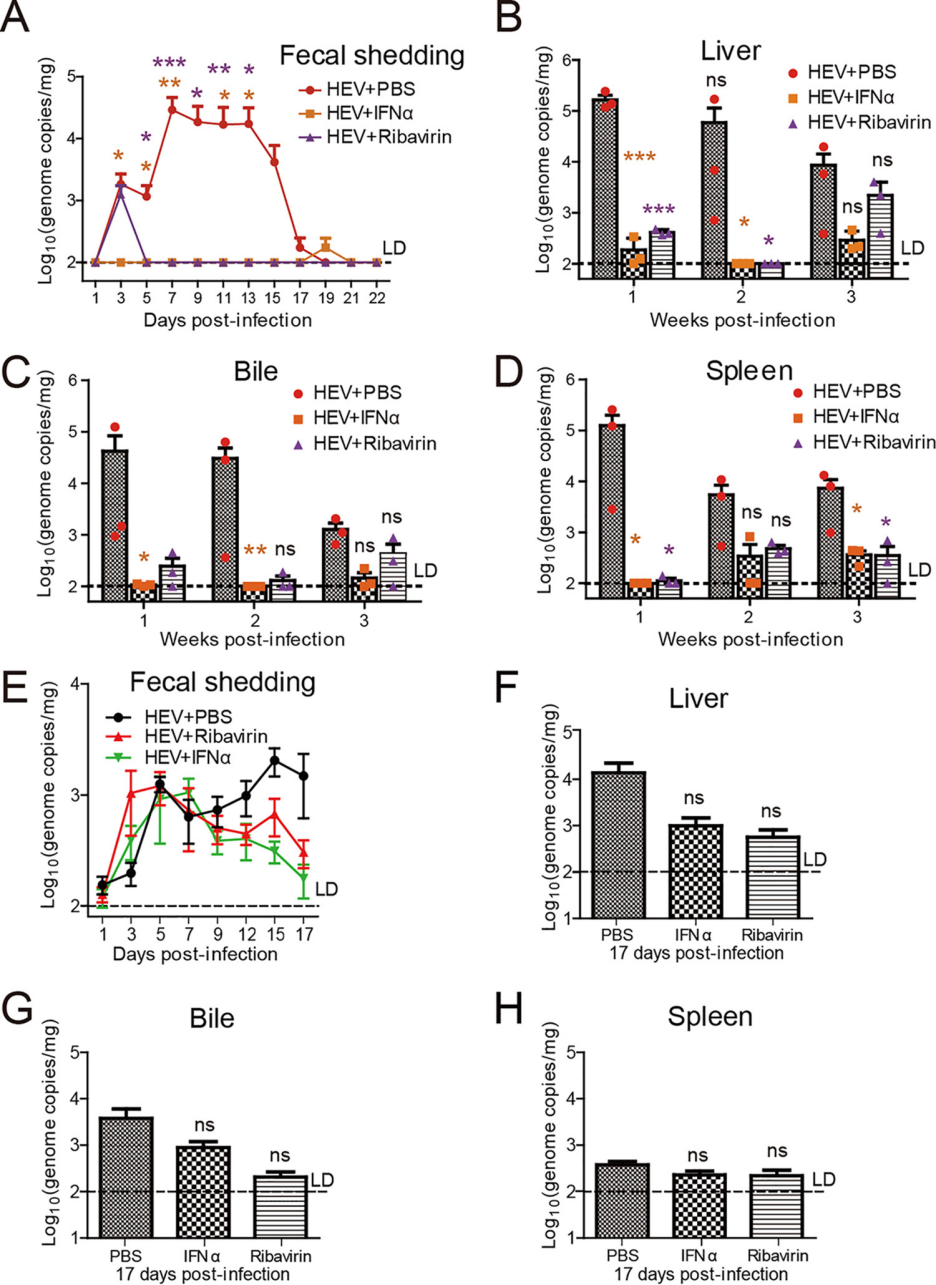
Assessment of the activities of ribavirin and peg-IFNα-2a of antiviral immunity in a gerbil model. Gerbils were infected intraperitoneally with infectious HEV p6 rescued from capped RNA transcripts from the P6 infectious cDNA clone. (A–D) Infected gerbils were subsequently (at 1 dpi) treated with PBS or different drugs: ribavirin (50 mg/kg/day) or peg-IFNα-2a (30 μg/kg/day) for 14 days, followed by 7 days without treatment before sacrifice. Viral RNA titers (genome copies/mg) in the feces, liver, bile, and spleen were measured at the indicated time points; *n* = 3 gerbils per group at each given time point. Ribavirin and peg-IFNα-2a treatment of HEV-infected gerbils delayed (A) fecal virus shedding and significantly reduced viral RNA load in (B) liver, (C) bile, and (D) spleen. (E–H) Infected gerbils were treated at 7 dpi with PBS, ribavirin (50 mg/kg/day), or peg-IFNα-2a (30 μg/kg/day) for 10 days before sacrifice. (E) Viral RNA titers in the feces were measured at the indicated time points; *n* = 5 gerbils per group at each given time point. HEV RNA loads in (F) liver, (G) bile, and (H) spleen were determined at 34 dpi by qPCR; *n* = 5 gerbils per group at each given time point. Error bars indicate standard deviation; *, *P *<* *0.05; **, *P *<* *0.01; and ***, *P *<* *0.001.

To further corroborate the utility of the HEV gerbil model for antiviral research, two antivirals ribavirin and peg-IFNα-2a, which have been validated by using the HEV p6-EZ replicon model in our previous study (36), were utilized, respectively (see “Animal experiment 4”). Since HEV replication was rapid as viral RNA could be detected as early as 3 dpi, three groups of i.p. infected gerbils were treated at 1 dpi with ribavirin (50 mg/kg/day) orally, peg-IFNα-2a (30 μg/kg/day) intraperitoneally, or with PBS. After 3 days of treatment, fecal virus shedding was significantly reduced in ribavirin-treated or peg-IFNα-2a-treated gerbils compared to PBS-treated control animals (Fig. 5A). Notably, only minimal level of HEV RNA was detected in the feces of ribavirin-treated or peg-IFNα-2a-treated gerbils from the 5th day of treatment (Fig. 5A). A decrease in viral RNA in the liver (Fig. 5B), bile (Fig. 5C), and spleen (Fig. 5D) was also observed upon administration of ribavirin and peg-IFNα-2a for 3 weeks.

To further confirm the effectiveness, the gerbils were postponed being treated with IFNα-2a and ribavirin at 7 dpi when HEV replication was reaching to the peak at this time point (Fig. 2A and B). A decrease in virus loads in the feces, liver, bile, and spleen was detected (but not significant), respectively (Fig. 5E to H). These data collectively demonstrate that the gerbil model provides a valuable tool for further HEV antiviral research.

### The gerbil model can be used for studying host antiviral sensing and responses.

**FIG 6:**
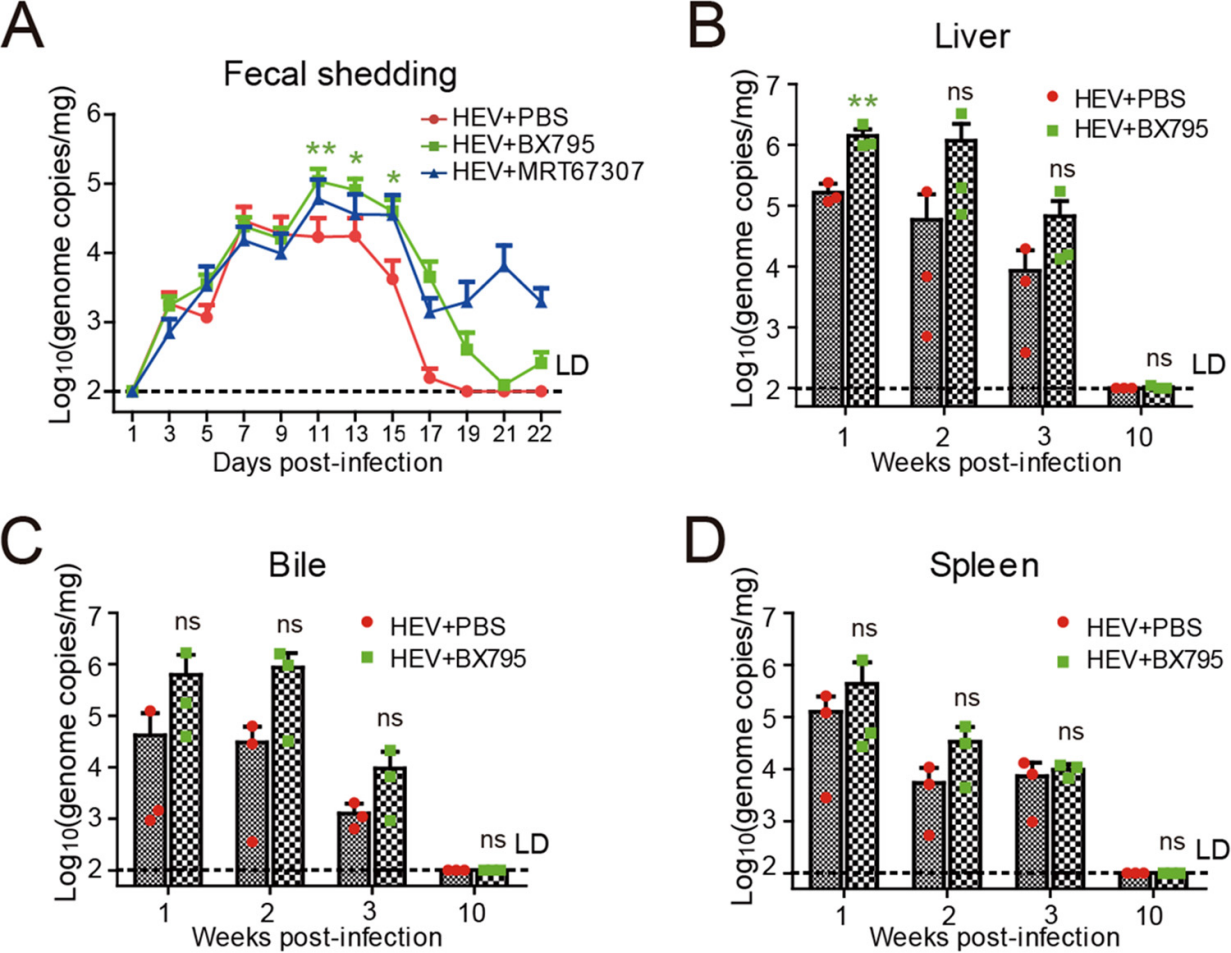
Assessment of the effects of TBK1 inhibitors on HEV replication in a gerbil model. Gerbils were infected with infectious HEV p6. Infected gerbils were subsequently (at 1 dpi) treated with PBS or under 10 weeks of continuous treatment with BX795 (30 mg/kg/day) or MRT67307 (30 mg/kg/day) before sacrifice. (A) BX795 and MRT67307 treatment increased fecal virus shedding. (B–D) BX795 was more effective in enhancing viral replication based on the amount of HEV RNA in the feces. BX795 treatment increased viral RNA load in (B) liver, (C) bile, and (D) spleen. Error bars indicate standard deviation; *, *P *<* *0.05; **, *P *<* *0.01; and ***, *P *<* *0.001.

In previous studies, we found that HEV replication was significantly enhanced *in vitro* when using two small molecule inhibitors targeting TANK-binding kinase 1 (TBK1) to block the RIG-I-like receptor (RLR)-IRF3 (interferon regulatory factor 3) pathway (36). To further demonstrate the utility of the HEV rodent model for studying host antiviral sensing and responses, we utilized the gerbil model to investigate the intriguing findings obtained from the persistent HEV replicon cell lines. Higher fecal virus shedding was observed in the groups treated i.p. with the TBK1 inhibitors BX795 (30 mg/kg/day) or MRT67307 (30 mg/kg/day) (Fig. 6A), accompanied by increased viral RNA levels in the liver, bile, and spleen of gerbils in the BX795-treated group in the first 2 wpi (Fig. 6B to D), but not in the MRT67307-treated group (data not shown). The result indicated that BX795 is more efficient than MRT67307 for promoting HEV replication by inhibition of TBK1 *in vivo*.

### Gerbils are susceptible to G4 but not G1 HEV infection following intrahepatic inoculation of full-length capped RNA transcripts.

**FIG 7:**
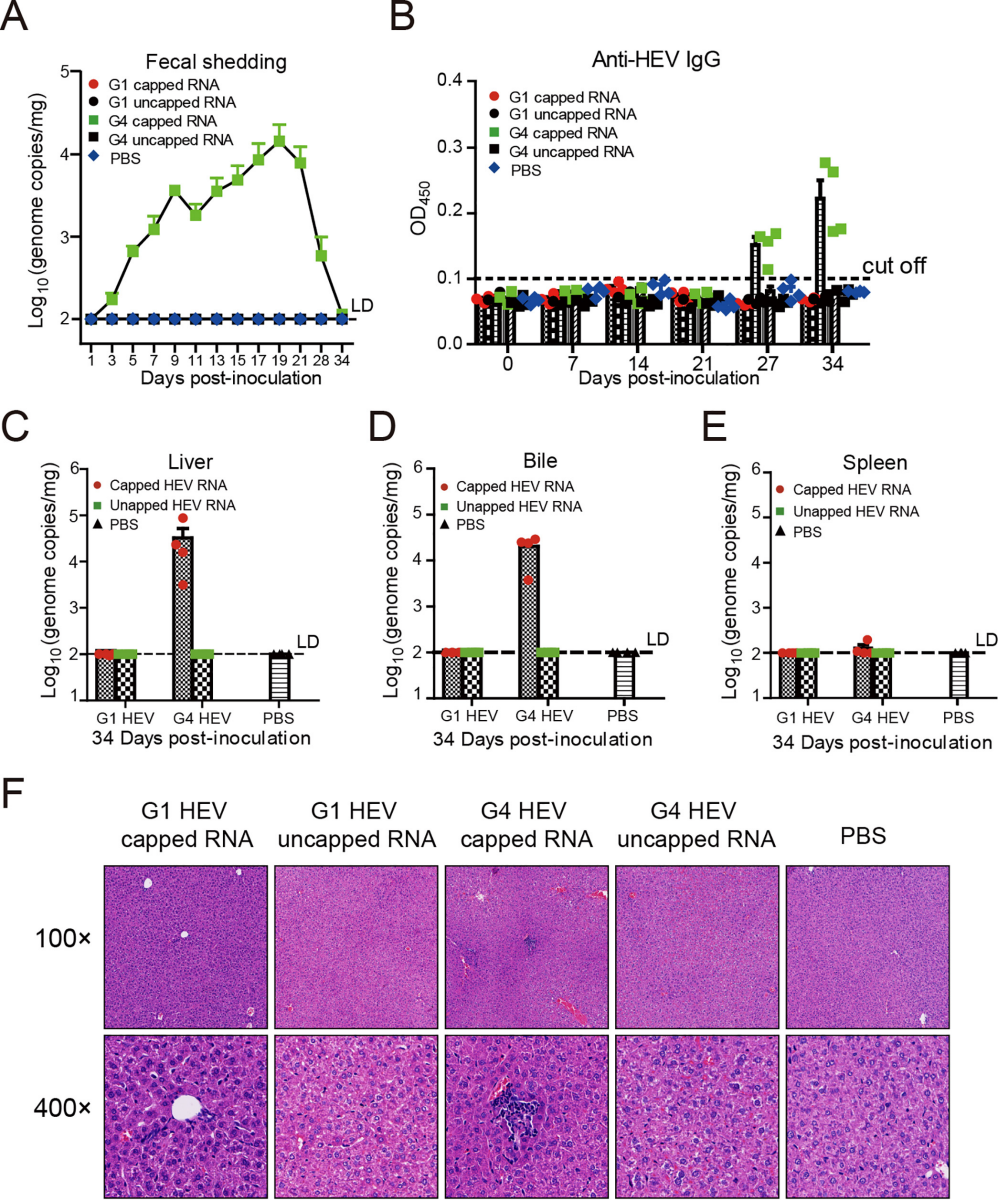
Assessment of the gerbil model for G1 or G4 HEV infection via intrahepatic inoculation of respective full-length capped HEV RNA transcripts. (A) Viral RNA in feces was determined by qPCR; *n* = 4 gerbils per group at each given time point. (B) Seroconversion to anti-HEV IgG in infected gerbils was detected by ELISA. The error bars in all samples indicate standard deviation. (C–E) HEV RNA loads in liver (C), bile (D), and spleen (E) were determined at 34 dpi by qPCR. (F) Liver sections from inoculated gerbils at 34 dpi, showing lymphocytic inflammatory infiltration in the G4 capped RNA transcripts group.

Inspired by the results from G3 HEV, we sought to test whether G1 or G4 HEV is indeed capable of infecting gerbils as well. Previously, the reverse genetics system for the G1 HEV Sar-55 strain and the G4 HEV TW6196E strain has been established, respectively (15, 37). However, since the yields of infectious virus stocks recovered in transfected cells were much lower for the G1 Sar-55 clone and the G4 TW6196E clone than for the G3 Kernow-C1 p6 (37, 38), the procedure of intrahepatic inoculation of HEV full-length genomic RNA transcripts was selected and carried out. *In vitro*-transcribed capped or uncapped RNA transcripts from G1 (Sar 55 strain) and genotype 4 (TW6196E strain) were inoculated into the liver of gerbils, respectively (“Animal experiment 5”). Fecal HEV RNA was only detected continuously in gerbils that received the capped G4 RNA transcripts, starting at 3 dpi and peaked at 19 dpi (Fig. 7A). Accordingly, all the gerbils in this group (*n* = 4) seroconverted to IgG anti-HEV antibodies at 27 dpi (Fig. 7B), and they each had detectable HEV RNA in the liver (Fig. 7C) and bile samples at 34 dpi (Fig. 7D). However, similar to the result of G3 RNA inoculum (Fig. 1B), G4 viral RNA was hardly detected in the spleen samples (Fig. 7E). Infiltration of lymphocytic inflammatory cells in the liver was observed by histological evaluation (Fig. 7F). In contrast, gerbils inoculated with uncapped G4 RNA transcripts, capped or uncapped G1 RNA transcripts, or PBS did not show significant signs of HEV infection (Fig. 7A to F). These results demonstrated that capped RNA transcripts from the G4 HEV clone, but not the G1 HEV clone, were infectious via intrahepatic injection of gerbils.

## DISCUSSION

A small animal model for HEV remains challenging. Nonhuman primates and pigs have been employed as effective animal models for HEV research, albeit with difficulty in handling and high costs (14–16, 25). With the discovery of HEV strains in various rodent species (39, 40), numerous attempts were made to develop a reproducible rodent model for HEV, but the results have been mixed thus far (14, 18, 23, 41). Chimeric mice with humanized livers have been reported (27, 42, 43), although the chimeric mice fail to reproduce the complex interactions between the innate pathway of the human cells and the adaptive immune pathway of the mice. The chicken HEV model had also been explored, but differences in chicken HEV genotypes from those that infect humans limit its broad use (21). A small conventional mammalian model for human HEV is still in great need for HEV antiviral development and pathogenesis study. In this study, we revisited and successfully developed a gerbil animal model for G3 human HEV, the virus genotype causing chronic hepatitis E in humans (44). We subsequently demonstrated the utility of this model in studying antivirals and host innate immune response, and verified that it could be applied to G4 HEV infection.

The Mongolian gerbil (Meriones unguiculatus) is a small rodent that had been used to study infection of coxsackievirus A16 and enterovirus 71 (45, 46). For HEV, several previous studies from the same laboratory in Beijing showed that infection of a G4 swine HEV from the liver samples led to detection of HEV antigens and lesions in various organs in gerbils (29, 31, 47, 48). However, we believe that whether these results were reliable and consistent should be validated by using biologically pure human HEV stocks generated by the HEV reverse genetics system and recognized standards of experimental procedures (15, 16, 18, 21). Our collaborators have developed infectious HEV cDNA clones of the G1 Sar-55 strain, G3 Kernow-C1 strain, and the G4 TW6196E strain, respectively, which are extensively used in many HEV research groups worldwide (15, 17, 37). Here we first demonstrated that intrahepatic injection of gerbils with capped G3 Kernow-C1 RNA transcripts resulted in HEV infection in four out of six animals that lasted up to 7 weeks (Fig. 1). Using the same approach, we also corroborated that G4 TW6196E RNA transcripts, but not G1 Sar-55 RNA transcripts, led to HEV infection in gerbils (Fig. 7). These results are consistent with the current knowledge that G1 HEV infect only humans or nonhuman primates, whereas G3 and G4 HEV are zoonotic, infecting both humans and specific animal hosts such as swine (3, 13, 14).

In order to establish an efficient gerbil model that is convenient to infect, we further inoculated gerbils intraperitoneally, which resulted in a robust HEV infection in each inoculated animal without the need for the use of transplanted human hepatocytes or immunosuppressive drugs (Fig. 2). The gross and microscopic liver lesions in infected gerbils (Fig. 3) are similar to those described in chicken HEV infection models (21), which makes the gerbil model very useful to characterize HEV infection *in vivo*. After seroconversion to HEV antibodies, the infected gerbils gradually stopped fecal virus shedding, and HEV RNA became undetectable in the bile, suggesting that virions are released via the biliary tract into the feces (35). Compared to HEV shedding in the bile and feces, the presence of HEV RNA in the liver and spleen tissues lasts longer. By using the gerbil model, we further demonstrated that HEV neutralizing antibodies and innate immunity play an important role in the antiviral response. However, oral inoculation is less efficient than the i.p. route for experimental HEV infection in gerbils (Fig. 4), likely due to resistance to the virus in the intestinal acidic environment and mucosal barriers.

It should be noted that using the Kernow-C1 p6 cloned virus with insertion of S17 in infection experiments is debatable, since the cloned virus may not reflect the feature of other wild-type G3 HEVs without S17 (49). However, the S17 insertion in the Kernow-C1 genome is not artificial; it was presented in a patient with chronic hepatitis E (50). Recent studies have shown that the insertion of S17 in ORF1 did not abolish HEV replication competency *in vitro* but also did not expand HEV host tropism *in vivo* (51). The Kernow-C1 strain shows a high replication efficiency *in vitro* (17, 50), which will help us to obtain sufficient HEV to inoculate animals. Due to the low replication efficiency of other HEV strains, it is difficult to collect enough viruses from cell cultures to inoculate gerbils. If the inoculated virus is isolated from the feces of infected patients or animals, the uncertainty in animal models increases. Therefore, the gerbil model infected with the Kernow-C1 strain has a high experimental stability and is easy to repeat. Another study had confirmed that the recombinant HEV with the S17 insertion did not gain an expanded infection capability in pigs (51). Moreover, when we used the G4 HEV (TW6196E) that does not contain the S17 sequence in this study, the gerbils also got infected efficiently (Fig. 7), suggesting that the S17 sequence in HEV is not crucial for infecting gerbils. Further comprehensive animal infection experiments are needed to test whether this rodent model applies to the non-S17-insertion G3 HEV.

Chronic hepatitis E with persistent HEV infection has recently become a significant clinical problem in immunocompromised individuals such as organ transplant recipients (13, 44, 52), and requires antiviral treatment. Unfortunately, due to the lack of a conventional HEV small animal model, HEV-specific drugs are currently lacking. Broad-spectrum antivirals such as pegylated IFN and ribavirin have been used clinically to treat hepatitis E with some successes (53, 54). However, these HEV-nonspecific drugs have severe side effects including transplant rejection and anemia (54). Ribavirin is a synthetic guanosine analogue that has shown antiviral activity against a range of DNA and RNA viruses, including hepatitis C virus and respiratory syncytial virus (55). To demonstrate the utility of the gerbil model for antiviral research, in this study, we showed the effectiveness of both peg-IFNα-2a and ribavirin in inhibiting HEV replication (Fig. 5). The results showed that the gerbil model is a useful tool for antiviral testing. Therefore, the availability of the model will facilitate future anti-HEV drug development efforts. Previously, we found that HEV replication was significantly enhanced *in vitro* when using small molecule inhibitors of TBK1 to inhibit host RLR-IRF3 phosphorylation (36). Here, we further corroborated, for the first time, that HEV replication was promoted to an extent by blocking the RLR-IRF3 pathway *in vivo* (Fig. 6), thus indicating that the gerbil model affords an opportunity to study host antiviral sensing and responses.

In conclusion, the conventional, low-cost gerbil HEV infection model opens a practical avenue for HEV research and will aid in future antiviral development and delineating mechanisms of host immune response.

## MATERIALS AND METHODS

### Infectious clones, virus stock and drugs.

The infectious cDNA clones of HEV G1 (pSK-HEV-2 from the Sar-55 strain) (15) and G3 (p6 from the Kernow-C1 strain) were generous gifts from Dr. Suzanne U. Emerson (NIH, Bethesda, MD), whereas the infectious cDNA clone of G4 (pHEV-4TW from the TW6196E strain) has been described previously (37). The Huh7-S10-3 cell line was also a gift from Dr. Emerson. S10-3 cells were maintained in Dulbecco's Modified Eagle Medium (DMEM) containing 10% FBS (Gibco), penicillin (250 IU/mL) and streptomycin (250 μg/mL) at 37°C in the presence of 5% CO_2_. MRT67307, BX795, and ribavirin were purchased from Selleck Chemicals, and peg-IFNα-2a was purchased from Shanghai Roche Pharmaceuticals.

The G3 Kernow-C1 HEV infectious virus stock used for gerbil inoculation was rescued from the p6 infectious cDNA clones in S10-3 cells as described previously (16, 36). The viral RNA titer of the rescued infectious HEV stock was determined by real-time qRT-PCR (56), with 6.83 × 10^6^ genome equivalents (GE) of viral RNA/100 μL medium, giving an infectivity of 1 fluorescent focus-forming unit (FFU)/5,618 GE, as determined previously (50).

### Immunofluorescence assay (IFA).

S10-3 cells transfected with HEV RNA transcripts or infected with HEV were grown on slides, rinsed in PBS three times, and fixed with 80% acetone for 20 min. Cells were then rinsed in PBS 3 times followed by 1-h incubation with anti-HEV-ORF2 antibody (36). Next, cells were rinsed in PBS for another 3 times and treated with goat antimouse IgG conjugated with Alexa Fluor 488 (Invitrogen) for 1 h. After adding 4, 6-diamidino-2-phenylindole (DAPI) mounting solution (Sigma), cells were visualized under a fluorescence microscope (DMI3000B, Leica, Germany). HEV infectious titers were assessed by counting the number of ORF2-positive cell clusters (quantified as FFU).

### Gerbils.

Specific-pathogen-free (SPF), male Mongolian gerbils of approximately 8–10 weeks of age were purchased from the Experimental Animal Center at the Zhejiang Academy of Medical Sciences (Hangzhou, China). Prior to inoculation, the animals were confirmed to be seronegative for HEV by a commercial ELISA kit (Wantai Biological Pharmacy Co., Beijing, China). All animal experiments were performed in strict accordance with the Experimental Animal Ethics Committee of Zhejiang University (IACUC approval no. ZJU20181049).

### Animal experiment 1: gerbils intrahepatically inoculated with RNA transcripts of the human HEV p6 (G3) cDNA clone.

Full-length capped or uncapped RNA transcripts were prepared from the p6 (Kernow-C1) HEV infectious cDNA clone using the mMESSAGE mMACHINE T7 ULTRA transcription kit (Ambion). *In vitro* transcription reactions were performed in a 60 μL reaction mixture containing 3 μg of linearized HEV plasmid DNA, and the purity and quality of the full-length RNA transcripts were verified by agarose gel electrophoresis as described previously (10). To determine the infectivity of G3 human HEV Kernow-C1/p6, 18 SPF gerbils (10 weeks old) were randomly assigned into three groups of six gerbils each. Gerbils were intrahepatically injected with full-length capped or uncapped HEV RNA transcripts (10 μL transcription reaction mixture diluted in 200 μl PBS for each gerbil) or equal amounts of phosphate-buffered saline (PBS) as controls. Briefly, the RNA transcripts were quickly thawed by addition of PBS and immediately injected into 4–5 different sites in the liver, with 40–50 μl per injection site. At 2 wpi (14 days) and 10 wpi, three gerbils were necropsied from each group, and samples of liver, bile, and spleen were harvested and tested for the presence of G3 HEV RNA by real-time RT-PCR. Fecal samples were tested for HEV RNA at different time points as shown in Fig. 1A, and serum samples were tested by ELISA for IgG anti-HEV antibodies weekly.

### Animal experiment 2: gerbils intraperitoneally inoculated with live G3 infectious HEV stock.

Briefly, 30 SPF gerbils were each inoculated intraperitoneally with 1 mL of the infectious virus stock containing approximately 6.83 × 10^7^ GE/mL. As negative controls, 30 gerbils were similarly inoculated with supernatants of Huh-7-S10-3 cells transfected with uncapped HEV RNA transcripts that were produced by repeated freeze/thaw cycles and centrifugation to clarify the cellular debris. Fecal samples were continuously collected from three given gerbils in each group at different time points and were tested for HEV RNA by real-time RT-PCR as shown in Fig. 2A. Six gerbils were necropsied at 70 days postinfection (dpi). At different time points as shown in Fig. 2B to D, three gerbils were necropsied from each group in each given time point. Serum samples were collected and tested by ELISA for anti-HEV IgG antibodies. Samples of liver, bile, and spleen were collected during necropsies and tested for HEV RNA. Gross lesions in the liver were examined at each necropsy. Histological examination and immunohistochemistry were conducted in liver sections from 2 to 4 wpi.

### Animal experiment 3: gerbils fed with live G3 infectious HEV stock orally.

Ten SPF gerbils were randomly assigned into 2 groups with five gerbils each. The five gerbils in the challenge group were each orally fed with 1 mL of the G3 infectious HEV stock containing approximately 6.83 × 10^7^ GE/mL. The remaining 5 gerbils in the control group each received 1 mL of DMEM. Fecal samples were collected and tested for HEV RNA at 1, 3, 5, 7, 9, 11, 13, 21, 27, and 34 dpi by real-time RT-PCR (Fig. 4A). Serum samples were tested for anti-HEV IgG antibodies weekly by ELISA. All the gerbils were sacrificed at 34 dpi. Samples of liver, bile, and spleen were collected during necropsies and tested for the presence of HEV RNA by real-time RT-PCR.

### Animal experiment 4: drug trials.

The gerbil model was used to test the effectiveness of known broad-spectrum HEV antivirals (peg-IFNα-2a and ribavirin) as well as TBK1 inhibitors (BX795, and MRT67307). Briefly, 54 SPF gerbils were inoculated intraperitoneally with 1 mL of the infectious virus stock containing approximately 6.83 × 10^7^ GE/mL. These HEV-infected gerbils were divided into five groups of daily treatments: orally treated with ribavirin (50 mg/kg/day; 9 gerbils) or treated via intraperitoneally with PBS (12 gerbils), peg-IFNα-2a (30 μg/kg/day; 9 gerbils), BX795 (30 mg/kg/day; 12 gerbils), or MRT67307 (30 mg/kg/day; 12 gerbils). The gerbils were treated with antivirals or TBK1 inhibitors at 1 dpi based on the results from animal experiment 2, in which the viral replication was rapid as HEV viral RNA could be detected as early as 3 dpi (Fig. 2A and B), and based on a previous study using rats as an infection model (18). HEV-infected gerbils were treated with ribavirin and peg-IFNα-2a for 3 weeks, whereas treatments with BX795 and MRT67307 were performed continuously for 10 weeks. In all 5 treatment groups, three gerbils were necropsied each week in the first 3 weeks. In the 10th wpi, the remaining three gerbils in the PBS, BX795, and MRT67307 groups were sacrificed. Samples of the liver, bile, and spleen of necropsied gerbils were tested for HEV RNA titers by real-time RT-PCR. Fecal samples were collected from the three gerbils that were necropsied at the last time point in each group, and tested for HEV RNA titers at different time points.

Alternatively, in the second drug trial, the gerbils were treated with IFNα-2a and ribavirin at 7 dpi (instead of 1 dpi) based on the results from animal experiment 2, in which the viral replication had reached to a high level at this time point (Fig. 2A and B). Briefly, 15 gerbils were inoculated intraperitoneally with live G3 HEV as described in experiment 2. At 7 dpi, these HEV-infected gerbils were randomly assigned into 3 groups of treatment: orally treated with ribavirin (25 mg/kg; 5 gerbils), or treated intraperitoneally with PBS (5 gerbils) or peg-IFNα-2a (15 μg/kg; 5 gerbils). HEV-infected gerbils were treated with antivirals for 10 days. All the gerbils were sacrificed at 17 dpi. Samples were collected and tested for HEV RNA titers as mentioned above.

### Animal experiment 5: gerbils intrahepatically inoculated with RNA transcripts of the human HEV Sar-55 (G1) or the TW6196E (G4) cDNA clone.

Full-length capped or uncapped HEV RNA transcripts from the Sar-55 or the TW6196E infectious cDNA clone were generated as described above. Twenty SPF gerbils were randomly assigned into 5 groups with four gerbils each. Gerbils in each group were intrahepatically injected with capped or uncapped G1 HEV RNA transcripts, capped or uncapped G4 HEV RNA transcripts (10 μL transcription reaction mixture diluted in 200 μl PBS for each gerbil), or equal amounts of PBS as controls, respectively. Fecal samples were collected and tested for HEV RNA at different time points shown in Fig. 7A. All the gerbils were sacrificed at 34 dpi. Serum samples were tested for anti-HEV IgG antibodies weekly by ELISA. Samples of liver, bile, and spleen were collected during necropsies and tested for the presence of HEV RNA.

### Histological examinations and immunohistochemistry (IHC).

For histological examinations, liver samples from infected and control animals were dissected, fixed in 4% paraformaldehyde for 12 h at 4°C, dehydrated in a graded series of ethanol, embedded in paraffin, and sliced into 6-μm-thick sections. The sections were subjected to histological examinations by hematoxylin and eosin (HE) staining. The anti-HEV-ORF2 antibody was used for IHC staining (36).

### ELISA, real-time qRT-PCR, and virus neutralization assay.

Serum and fecal samples were collected from all gerbils in animal experiments before and after inoculation as described above. At the end of the studies, serum samples were tested for anti-HEV IgG by a commercial ELISA kit (Wantai Biological Pharmacy Co., Beijing, China), and viral RNA in tissue samples and feces was detected by real-time RT-qPCR (25). Selected gerbil sera were tested in dilutions from 1:2 to 1:128 in a serum virus neutralization assay as described previously (57). The positive control sample Human-JS-1 is a convalescent phase serum with at least 10^4^ ELISA titer of anti-HEV IgG from an HEV-infected patient in Jiashan, Zhejiang province, China (58).

### Statistical analyses and reproducibility.

All the quantitative data are presented with dot and bar. Analyses of independent data were performed by Student’s unpaired two-tailed *t* test. Statistical analyses were carried out using GraphPad Prism 6.0. Differences were considered significant at *P* < 0.05. All samples, if preserved and properly processed, were included in the analyses, and no samples or animals were excluded. No statistical method was used to predetermine sample sizes, and gerbils in animal experiments were randomized to different groups.
